# Remarks on Animal Impregnation

**Published:** 1799-09

**Authors:** 


### *To the Editors of the Medical and Phyſical Journal.*

Gentlemen,

SHOULD the following remarks on the ſubject of Animal Impregnation, contain any thing in your opinion worthy of being ſubmitted to the public, your inſertion of them in the Medical Journal, will oblige

Your moſt obedient ſervant,

*July* 21, 1799. R. B. M.

Few ſubjects have oftener engaged the attention of the phyſiologiſt, than animal impregnation. From the time of Pythagoras, to the preſent, a number of theories have been preſented to us, many of which have been ingenious, and ſome very ridiculous. Among the number of thoſe which are now almoſt forgotten, I think that of Harvey merits notice; and the more ſo, as I think it bears a conſiderable ſimilitude to the opinion advanced by Dr. Haighton, in a very ingenious paper publiſhed in the "*Philoſophical Tranſactions*," Part I. 1797.—There have been few phyſiologiſts, except Harvey and Dr. Haighton, who have not ſuppoſed that the rudiments of the fœtus exiſted in the male ſemen; and it is well known, that the theory ſupported by Lieuwenhoek ſuppoſed that the fœtus in miniature, endued with animal life, was actually contained in the male ſemen

ſemen.—This hypotheſis, ridiculous as it may now appear, was greedily and almoſt univerſally received at one time; a ſtriking proof of the fondneſs for the marvellous, to which human nature is ſo prone, and which is ſo injurious to the cauſe of truth.

Bidding adieu to theſe idle opinions, I will now take the liberty of making a few remarks on the theory propoſed by Dr. Haighton.

It appears from the reſult of Dr. Haighton's well-directed experiments, that the formation of *corpora lutea* in the ovaria is the true teſt of impregnation having happened, and that the contact of the ſemen with the ovaria is not neceſſary to produce this effect; which is aſcribed to *ſympathy or conſent of parts*.—This expreſſion, however, does not perhaps convey a very clear idea of the ſubject. Might I be allowed to turn commentator, I ſhould ſay, that the act of coition induces impregnation, and that the ſemen paſſing into the vagina, is thence abſorbed, and carried into the general ſyſtem, where, by its peculiar ſtimulus, it produces thoſe changes, which happen after impregnation, in the uterus, its appendages, and the breaſts; ſo that I conſider the ſtimulus of the ſemen as perfecting what the ſtimulus of coition had begun.

Whether my comment will ſerve to elucidate the original, remains for others to determine; but before I conclude, I ſhall endeavour to obviate one objection which may be made to this theory.—It may be ſaid, if impregnation is occaſioned merely by a ſtimulus, how is it that females never conceive from any cauſe, except the introduction of ſemen into the vagina? This query, however, would hardly be conſidered as a difficulty by an abettor of the theory, and I ſhould not have noticed it, had it not afforded me an opportunity for mentioning a remarkable change which ſometimes takes place in the ovaria; and which I think affords ſupport to Dr. Haighton's theory. In anſwer, then, to the above-mentioned query, I would ſay, that proper conception can take place from no ſtimulus except ſemen, yet that ſome of the changes conſequent to impregnation, may, and do ſometimes happen, from ſtimuli of other kinds. It is well known to thoſe who are in the habit of diſſecting, that hair, teeth, and other fœtal remains, are ſometimes found in the ovaria, and ſome appearances of this kind have been obſerved in ſubjects, where it was evident that impregnation could not have taken place from coition. This circumſtance led Mr. Hunter to explain the growth of hair in this ſituation, by ſaying, "that the ovarium had taken on a cuticular ſtructure;" but I think that it is much more eaſily and rationally explained, by ſuppoſing that in thoſe caſes, a very imperfect kind of impregnation had taken place, from ſome unuſual ſtimulus; and it will, perhaps, be found, that ſome other diſeaſed

 alteraalterations occafionally happening in the female fyftem, may with propriety be referred to the fame caufe.

Thofe remarks are fubmitted to the public merely as conjectures, which if they are not perfectly juft, may affift in the attainment of truth by promoting inquiry.

